# Artificial Intelligence-Assisted CRISPR/Cas Systems for Targeting Plant Viruses

**DOI:** 10.3390/genes16111258

**Published:** 2025-10-24

**Authors:** Nurgul Iksat, Almas Madirov, Kuralay Zhanassova, Zhaksylyk Masalimov

**Affiliations:** Rustem Omarov Plant Biotechnology Laboratory, Department of Biotechnology and Microbiology, L.N. Gumilyov Eurasian National University, Astana 010008, Kazakhstan

**Keywords:** CRISPR, plant virus, artificial intelligence, resistant plants, genome editing efficiency

## Abstract

Plant viral infections continue to pose a significant and ongoing threat to global food security, especially in the context of climatic instability and intensive agricultural practices. The CRISPR/Cas system has emerged as a powerful tool for developing virus-resistant crops by enabling precise modifications to viral genomes or plant susceptibility factors. Nonetheless, the efficacy and dependability of CRISPR-based antiviral approaches are limited by challenges in guide RNA design, off-target effects, insufficiently annotated datasets, and the intricate biological dynamics of plant–virus interactions. This paper summarizes the latest advancements in the incorporation of artificial intelligence (AI) methodologies, including machine learning and deep learning algorithms, into the CRISPR design and optimization framework. It examines how convolutional and recurrent neural networks, transformer architectures, and generative models like AlphaFold2, RoseTTAFold, and ESMFold can be used to predict protein structures, score sgRNAs, and model host–virus interactions. AI-enhanced methods have been proven to improve target specificity, Cas protein performance, and in silico validation. This paper aims to establish a foundation for next-generation genome editing strategies against plant viruses and promote the adoption of AI-powered CRISPR technologies in sustainable agriculture.

## 1. Introduction

Despite significant improvements in breeding and agricultural technologies, viral plant diseases remain one of the most devastating biotic factors, severely constraining the productivity and quality of agricultural crops worldwide. The Food and Agriculture Organization of the United Nations estimates that yearly output losses in tropical and subtropical regions from viral infections can exceed 30%, particularly in crops lacking inherent resistance [1]. Viruses such as tomato yellow leaf curl virus (TYLCV), rice tungro bacilliform virus (RTBV), cotton leaf curl virus (CLCuV), and maize streak virus (MSV) pose major threats to important food crops. Host species such as *Solanum lycopersicum*, *Oryza sativa, Gossypium hirsutum* and *Zea mays* are particularly vulnerable due to the high virulence and rapid spread of viral pathogens via vectors, seeds, or mechanical transmission [2,3,4,5,6]. The intricacy and adaptability of viral infections, limited protection strategies, and the deficiency of resistant variations necessitate the advancement of novel, high-precision biotechnological solutions. The CRISPR/Cas system, derived from the adaptive immune response of bacteria, is one of the most promising genome editing platforms, reconfigured as a universal tool for precise manipulation of DNA and RNA [7,8,9,10,11]. In plant science, CRISPR/Cas is employed for the direct annihilation of viral genomes and for the alteration of host plant genes associated with the detection and dissemination of infection [12]. Over the past decade, nucleases like Cas9, Cas12a, and Cas13a, which are efficient against many kinds of viruses, encompassing both DNA and RNA viruses, have been successfully modified and utilized [13,14,15,16]. The efficacy of CRISPR systems in plants is largely contingent upon the appropriate selection of essential components: guide RNA (gRNA) [17,18], a variant of the Cas protein with the requisite biochemical properties [19,20], and delivery mechanisms [21,22]. However, precise targeting is challenged by the rapid mutation rates of plant viruses and limited functional annotation of susceptibility genes, especially in under-studied crop species. These constraints hinder the broad applicability of conventional genome editing strategies.

Given these challenges, the application of artificial intelligence (AI) techniques, encompassing machine learning (ML) [23,24,25] and deep learning (DL) [26,27,28], for the analysis, modeling, and optimization of CRISPR editing is especially significant. Contemporary AI models utilizing convolutional neural networks (CNNs) [29,30,31], recurrent neural networks (RNNs) [31,32,33], and transformer [34,35] architectures exhibit significant precision in forecasting gRNA efficiency and specificity, detecting off-target effects, and examining interactions between viral and plant proteins [36,37,38]. Initial research on the in silico design of Cas proteins exhibiting certain attributes, including enhanced PAM (Protospacer Adjacent Motif) specificity, stability inside plant cells, and modularity of functional domains, is emerging. Nonetheless, the majority of these models exhibit restricted transferability among plant species, particularly between model organisms and agricultural crops characterized by extensive and intricate genomes.

The necessity to analyze and integrate multi-omics data from genomes, transcriptomics, proteomics, and metabolomics adds an extra layer of complexity. This data serves as a crucial resource for discovering resistance variables, modifying targets, and forecasting intervention efficacy. An instance is the transcriptome analysis of *A. thaliana* infected with tobacco etch virus (TEV), which discovered differentially expressed immune response genes, such as PR1 and EDS1, linked to the salicylate-dependent defense system [39]. These data facilitated high-accuracy classification of resistant and susceptible genotypes only after their incorporation into a machine learning model. An analogous methodology was employed in the examination of *Solanum lycopersicum*’s reaction to tomato spotted wilt virus (TSWV), wherein gradient boosting discerned pivotal genes (WRKY, MYB, EDS1) with a classification accuracy of 89% [40]. It is important to acknowledge that many models are trained on limited datasets and often fail to include cross-validation or a distinct division between training and testing sets, resulting in overfitting and inflated accuracy estimates. The use of computer vision techniques in combination with deep learning architectures such as convolutional neural networks (CNNs) has enabled automated phenotypic analysis of viral symptoms, thereby advancing disease diagnostics. A hybrid CNN and Random Forest model attained an accuracy of up to 95% in assessing the extent of damage to Nicotiana tabacum leaves infected with tobacco mosaic virus (TMV) in photos subjected to varying lighting conditions and morphologies [41,42]. Moreover, GWAS and pan-genome analysis techniques provide avenues for the precise identification of resistance alleles. A pan-genome investigation of 3000 *Oryza sativa* genomes revealed polymorphisms in eIF(iso)4G and RYMV1 linked to resistance against RTBV and rice yellow mottle virus (RYMV), which were functionally validated via CRISPR interference [43,44]. Despite these advances, several challenges persist, including the absence of cohesive annotated datasets, the inadequate portability of AI models developed on human-derived data, and the limited interpretability of numerous algorithms. Moreover, regulatory concerns and bioethical evaluations of genome-edited plants, especially those employing AI, necessitate thorough analysis and discourse.

**Figure 1:**
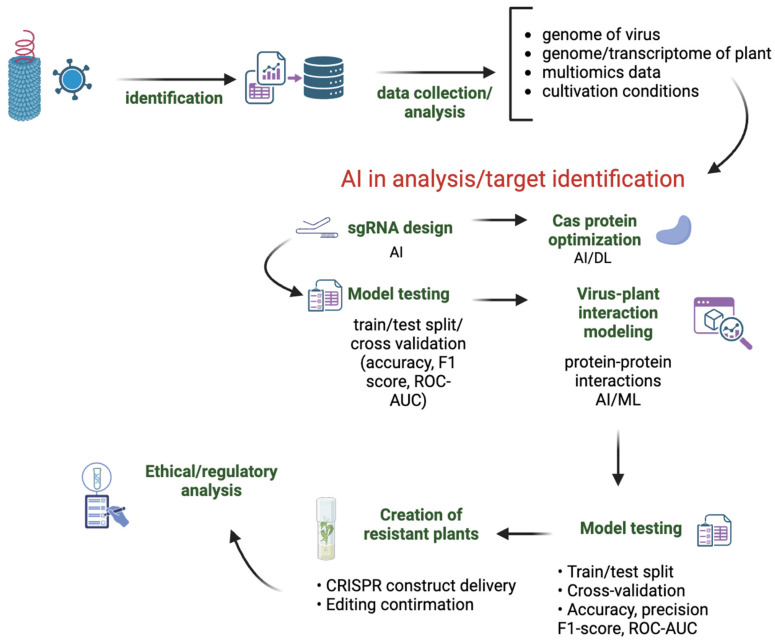
Schematic overview of the application of artificial intelligence (AI) and machine learning (ML) in the development of virus-resistant plants using CRISPR-based genome editing. The process begins with the identification of the virus and proceeds to comprehensive data collection and analysis, including the viral genome, host plant genome/transcriptome, multi-omics data, and cultivation conditions. AI and deep learning (DL) methods are employed for Cas protein optimization, modeling virus–plant protein–protein interactions, and designing sgRNAs. These models are validated through train/test splitting and cross-validation techniques, using performance metrics such as accuracy, F1 score, and ROC-AUC. The selected sgRNAs and optimized CRISPR constructs are delivered to plants, followed by confirmation of successful genome editing. The final step involves the creation of resistant plants and ethical/regulatory analysis to ensure compliance with biosafety and bioethics standards.

This review paper aims to examine the potential and existing applications of artificial intelligence technologies in CRISPR/Cas systems for the protection of plants against viral infections. It investigates the role of AI in the design of guide RNAs, the engineering of Cas proteins, and the modeling of virus–plant interactions. In addition, the review outlines emerging research directions and discusses regulatory considerations relevant to AI-assisted genetic engineering (Figure 1).

## 2. AI-Enhanced CRISPR Strategies Against Plant Viruses

### 2.1. AI/ML Tools and Algorithms for sgRNA Design

CRISPR/Cas technologies have become essential to plant biotechnology, serving as a pivotal instrument for precise genome editing. Nonetheless, a considerable disparity persists between effective experimental applications and the advancement of computational techniques for the systematic design of guide RNAs (sgRNAs) [45,46]. The principal constraint continues to be the exceedingly scarce quantity of specialist models tailored for plant systems. Efforts to use algorithms derived from mammalian or prokaryotic data frequently result in a significant decline in predictive accuracy, underscoring the distinctiveness of plant genomes and the necessity for the creation of indigenous solutions [47,48,49]. This issue is especially evident in crops with extensive and intricate genomes, such as wheat and maize, where repetitive elements and atypical PAM motifs are prevalent [50,51]. The disparity is more obvious when juxtaposed with medical genomic engineering, where numerous precise and advanced machine learning (ML) methods, founded on vast in vivo and in vitro datasets, have been established in recent years [52,53].

In plant biology, a notable effective solution is sgRNACNN [54,55,56], an ensemble of convolutional neural networks trained on in planta data for four crops: *A. thaliana*, *Oryza sativa*, *Zea mays*, and *Solanum lycopersicum*. The model exhibits a 15–30% enhancement in accuracy for forecasting guideline performance vs. current universal methods, but solely inside the initial training area [57,58]. This illustrates the overarching issue of inadequate transferability of AI models among crops, particularly in the absence of transfer learning, fine-tuning, or few-shot learning. Less complex yet interpretable positional classifiers, exemplified by the model of Das et al. (2023) [59], were utilized on *Capsicum annuum* and *N. benthamiana*, yielding moderate accuracy (correlation coefficients of 0.45–0.55) while demanding minimal computational resources, thus appealing to laboratories with constrained infrastructure. The benefit of these models lies in their predictive explainability, enabling the identification of nucleotide locations and factors that affect sgRNA efficiency. Their primary limitation is their failure to consider the spatial (3D) architecture of the genome and epigenetic changes, which significantly influence gene expression in plants.

Commonly utilized general-purpose tools, including DeepCpf1, CRISPR-HNN, sgRNA Scorer 2.0, TIGER, CHOPCHOP, E-CRISP, CRISPR Genome-wide Analysis, and Cas-OFFinder, were predominantly designed for humans and bacteria [60]. For instance, DeepCpf1, a model based on deep neural networks for predicting Cas12a (Cpf1) activity, demonstrates elevated ROC-AUC (>0.8) and R^2^ (>0.6) in human systems [61]. Nonetheless, when implemented using in planta data in rice, the accuracy diminishes by 25–30%, attributable to both biological discrepancies and diversity in PAM preferences and Cas12a sensitivity to sequence context in plants [30,62]. The CRISPR-HNN model, a hybrid design integrating convolutional and recurrent neural networks, demonstrates exceptional efficacy in extracting both local and global features [63,64]. The absence of plant data during training results in prediction instability when analyzing extremely complex genomes, such as those of wheat and maize, characterized by prevalent repetitions and intricate spatial structures [65]. Nevertheless, the majority of research fails to reveal validation criteria, like the type of cross-validation employed, the proportion of the test sample utilized, or the incorporation of independent datasets. This diminishes the likelihood of reproducibility and objective evaluation of prediction quality.

Among traditional but common approaches, sgRNA Scorer 2.0 is prominent. Originally designed as a general-purpose instrument, it has been predominantly evaluated using human and murine data; however, it has also been effectively employed in gene editing endeavors involving rice and tobacco, notably in the development of resistance to tobacco mosaic virus (TMV) [66,67]. TIGER, CHOPCHOP, and E-CRISP are extensively utilized. These tools offer user-friendly web interfaces and facilitate the swift creation of lists of prospective sgRNAs, including assessments of off-target effects. Nonetheless, the majority of these are founded on scientific principles and statistical models obtained from animal or prokaryotic data. Their prediction capability in plant genomes is constrained. For instance, correlation coefficients with trial outcomes never surpass 0.3–0.4, and the compilation of suggested guidelines frequently encompasses locations that are ineffectual in practice. Approaches to sgRNA design guided by AI must move beyond sequence-level parameters and incorporate a deeper understanding of viral evolutionary constraints. In the context of geminiviruses, direct CRISPR/Cas-mediated interference is most successful when targeting highly conserved genomic regions, such as the intergenic region (IR) and the replication-associated protein gene (Rep). By contrast, targeting more variable open reading frames, particularly those encoding capsid proteins (CPs), frequently results in reduced editing efficiency due to the rapid emergence of escape variants. This phenomenon has been experimentally confirmed. Ali et al. (2021) [68] demonstrated that CRISPR/Cas9 constructs directed at the IR of several geminiviruses resulted in robust and stable viral interference in *Nicotiana benthamiana*. In the same study, constructs targeting the CP gene permitted partial viral replication and gave rise to escape mutants. Similarly, Tripathi et al. (2021) [69] reported that targeting the Rep gene of African cassava mosaic virus (ACMV) conferred stable resistance in transgenic cassava lines, whereas CP-targeting sgRNAs showed lower efficacy and editing persistence.

Beyond geminiviruses, targeted CRISPR/Cas interventions have also been explored in other plant-infecting DNA and RNA viruses. In tomato, Ali et al. (2015) [70] also successfully reduced TYLCV accumulation by targeting both the IR and ORF C1. These findings reinforce the strategic advantage of multiplex targeting to mitigate viral evasion. Maree et al. (2010) [71] characterized a cluster of subgenomic RNAs encoding ORFs 3–12 in grapevine leafroll-associated virus 3 (GLRaV-3), highlighting the need for precision in selecting target sites in large 3′-terminal ORF arrays. Efforts to develop broadly effective sgRNAs have included the use of AI-informed models trained on viral diversity. Liu et al. (2021) [72] reported the design of low-strain-specificity sgRNAs targeting conserved regions of the ORF1 (RdRP) across multiple *Potyvirus* species. Similarly, computational strategies have been applied to design sgRNAs targeting ORF6 and ORF9 of beet yellows virus (BYV) [73], providing proof-of-concept for CRISPR-based disruption of functional viral modules. Importantly, these challenges are not limited to DNA viruses. RNA viruses such as tobacco mosaic virus (TMV) and cucumber mosaic virus (CMV), characterized by high mutation rates, pose a unique difficulty for stable CRISPR targeting. Here, ML models offer a valuable solution by identifying evolutionarily constrained and functionally essential regions across viral genomes. As emphasized by Zaidi et al. (2020) [74], conservation-guided sgRNA design through AI can reduce the likelihood of viral escape, particularly when combined with multiplexed editing strategies. Collectively, these findings underscore that effective CRISPR-mediated resistance requires thoughtful selection of target loci prioritizing conserved, replication-related ORFs such as Rep and RdRP over structurally plastic genes like CP. The integration of AI tools into this process enables a more nuanced and predictive approach to sgRNA design, which is essential for achieving durable viral resistance in crops and for advancing our mechanistic understanding of plant–virus interactions.

TIGER provides efficient batch processing of targets; however, its heuristics neglect the distribution of PAM motifs and the prevalence of pseudogenes in plant genomes, resulting in a significant proportion of false positives [75]. CHOPCHOP and E-CRISP are favored for their user-friendliness and compatibility with several Cas systems in rice, tomato, and *A. thaliana*. Specifically, CHOPCHOP was utilized with tomato leaf curl virus (ToLCV), illustrating the tool’s adaptability [76]. Nonetheless, their algorithms for evaluating on-target activity rely on rudimentary measures such as GC composition and the existence of certain motifs, which are not tailored to plant data. While they may provide a foundation for researchers engaged in preliminary initiatives, their predictions necessitate compulsory experimental validation for essential applications. The Cas-OFFinder tool has been utilized to evaluate off-target effects in genome editing initiatives for soybeans, corn, and tobacco, in addition to the creation of CRISPR-based diagnostic systems targeting viral RNAs [77,78]. This tool remains widely used due to its speed and compatibility with various Cas nucleases; however, it performs only mechanical sequence enumeration and fails to consider the biological context. This may result in an exaggerated assessment of risks and the inundation of experiments with superfluous candidates. Conversely, CRISPR Genome-wide Analysis facilitates extensive screening over the entire genome; nevertheless, it is hindered by an absence of plant-specific filters and training data [79,80,81].

A comparative review of current methods reveals that the primary restriction resides not in the computational architecture, but in the insufficient systemic adaptation to plant data. Even very proficient models like DeepCpf1 or CRISPR-HNN, when provided with high-quality in-plant datasets, might attain correlation values of 0.7 or above. Nevertheless, due to the current scarcity of such data, their predictive accuracy is comparable to random selection of candidates based on marginal or non-informative features.

The existing ecosystem of prediction tools for CRISPR/Cas in plants comprises a limited selection of specialized models and numerous general-purpose solutions with reduced transferability [82] (Table 1). To surmount existing constraints, the following are requisite: Systematic aggregation of extensive in-plant datasets; formulation of adaptive machine learning models derived from this data; and establishment of hybrid solutions that integrate the precision of deep learning with the comprehensibility of conventional methodologies.

### 2.2. Modeling and Optimization of Cas Proteins Using AI

The engineering of Cas proteins for the suppression of plant viruses is a contemporary focus in plant biotechnology, wherein artificial intelligence techniques, especially deep learning, exhibit significant promise for the rational design of proteins with enhanced specificity, efficacy, and controllability. In contrast to the extensively examined selection of guide RNAs, the creation of Cas proteins necessitates comprehension of their molecular characteristics, the architecture of their interactions with nucleic acids, and their functionality under particular plant cell circumstances [24,83]. Deep learning facilitates the modeling of these factors utilizing extensive biological data, encompassing amino acid sequences, three-dimensional structures, and interaction information with viral genomes [84,85].

The initial systematic application of AI-assisted CRISPR technology to combat plant viral infections was reported by Zhang et al. (2018) [86], who employed a modified FnCas9 endonuclease from *Francisella novicida* to inhibit cucumber mosaic virus (CMV) and tobacco mosaic virus (TMV) in *Nicotiana benthamiana* and *Arabidopsis thaliana*. In this study, the CRISPR/Cas components were delivered using a tobacco rattle virus (TRV)-based vector system, enabling systemic expression *in planta*. The sgRNAs were designed to specifically target the coat protein (CP) genes of CMV and TMV, which are essential for virion assembly and systemic movement. The selection of effective FnCas9 variants was guided by convolutional neural network models trained to predict RNA-protein interaction interfaces, enhancing binding affinity and cleavage efficiency. This AI-guided optimization contributed to a marked reduction in viral load, and importantly, the antiviral effect was shown to be heritable in subsequent plant generations. Another instance was the SpCas9 initiative targeting the cotton leaf curl Multan virus (CLCuMuV) conducted by Yin et al. (2019) [87]. Algorithms utilizing ensemble decision trees and a neural network classifier were employed to identify two extremely effective Cas9-targeted locations in viral DNA. While a traditional delivery strategy was employed throughout the in-plant phase, the protein selection stage and activity prediction relied on AI analysis of nuclease activity inside the framework of the viral genome. This technique guaranteed total viral resistance in *N. benthamiana* specimens.

In the case of RNA viruses like TMV, Cas13 family proteins have demonstrated significant utility. Cao et al. (2021) [88] employed the CasRx enzyme (RfxCas13d), characterized by its single-stranded RNA cleavage capability, to target foreign RNA viruses. Attention-based deep learning algorithms were employed to examine the secondary structure of viral RNA and pinpoint areas of greatest accessibility. The application of CasRx to *N. benthamiana* plants resulted in decreased viral expression and observable symptoms of infection. One significant advantage of CasRx is its diminutive size and autonomy from PAM or PFS, which facilitates engineering. Nonetheless, CasRx activity is contingent upon the cellular environment, and in certain instances, the HEPN domain may induce non-specific RNA cleavage, particularly when expression is aberrant.

Prior research by Ji et al. (2015) [89] employing SpCas9 to target beet severe curly top virus (BSCTV) exemplifies one of the initial instances of genetically engineering viral resistance by direct modification of the viral genome. Artificial intelligence was employed in the selection of the most conserved locations in viral DNA, utilizing techniques such as the positional weight model and logistic regression analysis. The study was conducted in *N. benthamiana* and *A. thaliana*, achieving a significant decrease in viral replication. Ghorbani Faal et al. (2020) [90] utilized inducible Cas9 expression to inhibit tomato yellow leaf curl virus (TYLCV) in Solanum lycopersicum. AI was employed to model promoter designs and forecast the appropriate temporal progression of Cas9 activation upon infection. This method facilitated virus-specific activation of editing with few side effects. Nonetheless, the majority of models employed for Cas protein design do not incorporate in-plant data and are restricted to in vitro or in silico simulations. This establishes a substantial disparity between computational forecasts and actual biological efficacy under host plant conditions.

Consequently, deep learning is emerging as a crucial instrument in the design of Cas proteins with enhanced characteristics aimed at specific plant viruses (Table 2). This encompasses activity forecasting, optimization of target interactions, creation of novel protein variations, and adaptation to plant physiology. Principal advantages encompass elevated accuracy, capacity to study hitherto unexamined proteins, and integration with in situ testing. Constraints encompass data prerequisites and model interpretability; however, the swift advancement of structural frameworks and explainable AI algorithms is facilitating the mitigation of these obstacles.

In addition to direct antiviral strategies, AI has shown growing potential in identifying and modifying plant host susceptibility (S) genes and immune regulators to enhance resistance against viral pathogens. One of the most studied targets is the *eIF(iso)4G* gene, a translation initiation factor exploited by viruses such as rice tungro bacilliform virus (RTBV) and rice yellow mottle virus (RYMV). Using a combination of pan-genome association studies, SNP-effect prediction, and structural modeling via AlphaFold2, researchers successfully identified resistance-associated polymorphisms in *eIF(iso)4G*, which were functionally validated using CRISPR interference in *Oryza sativa* [91]. Similarly, the receptor-like kinase *NIK1*, known to inhibit viral replication through translational shutdown, was targeted by CRISPR/Cas systems guided by AI-driven co-expression network analysis (WGCNA) and motif discovery tools. These approaches enabled the precise editing of *NIK1* regulatory regions to enhance antiviral immunity without compromising plant development [87,92]. Another promising target is *BAK1* (BRI1-ASSOCIATED KINASE 1), a key component of pattern recognition receptor complexes. AI-based protein–protein interaction predictors, such as DeepInteract and AlphaFold-Multimer, have been employed to map interactions between *BAK1* and viral effectors, thereby informing CRISPR-based strategies to alter viral binding sites while preserving core immune function [93]. In the context of RNA metabolism, DEAD-box helicases such as *RH20* have been identified as crucial host factors supporting the replication of RNA viruses. Through transcriptomic analysis, Random Forest classifiers, and gene regulatory network modeling (GENIE3), *RH20* homologs were recognized as central hubs and subsequently edited using CRISPR/Cas12a to suppress viral proliferation in *Nicotiana benthamiana* [94,95]. These findings collectively demonstrate how AI-integrated methodologies, including structural prediction, network inference, and machine learning classifiers are becoming instrumental in guiding CRISPR-based interventions aimed at host determinants of viral susceptibility. The strategic targeting of plant factors such as *eIF(iso)4G*, *NIK1*, *BAK1*, and DEAD-box helicases opens up new avenues for durable and broad-spectrum resistance engineering in crops.

### 2.3. Machine Learning in the Analysis of Virus–Host Interactions

The application of machine learning (ML) techniques in examining molecular interactions between plants and viruses is a very promising and swiftly advancing domain within molecular phytopathology [96,97,98]. These interactions are fundamental to viral pathogenesis and predominantly entail protein–protein interactions between viral effector proteins and host cellular elements that regulate replication, intracellular transport, and immunological suppression [99,100]. Due to the intricacy of these interactions and the scarcity of experimental data, machine learning algorithms serve as a valuable instrument for their systematic study, enhancing the ability to forecast plant resistance and pinpoint targets for precise genome editing.

An illustrative instance of a particular interaction between viral and plant proteins is the binding of the βC1 protein, encoded by the satellite RNA of the cotton leaf curl virus Multan betasatellite (CLCuMB), to the plant E2 conjugase of the UBC3 ubiquitin system in *Gossypium hirsutum* [101]. This interaction interferes with proteasome-mediated protein degradation and undermines the plant’s antiviral defenses. Kamal et al. (2019) [102] conducted energy modeling of this complex utilizing molecular dynamics and machine learning techniques, enabling the identification of putative inhibitory peptides that can obstruct the interaction.

Contemporary models for predicting protein–protein interactions (PPI) between viruses and plants generally employ vectorization of amino acid sequences (such as AAC, DPC, PAAC descriptors), with structural and physicochemical attributes [103,104,105]. The data serve as input characteristics in supervised models that are trained on restricted interaction sets and subsequently calibrated on new data. Zhang et al. (2024) [106] introduced the CBIL-VHPLI model, which integrates a hybrid convolutional neural network (CNN) with a bidirectional long short-term memory (BiLSTM) architecture, employing transfer learning techniques. The model exhibited excellent accuracy of 91.6% and a precision of around 93% when evaluating the interactions between the βC1 protein and UBC3 in *Gossypium hirsutum*. Nonetheless, taking sgRNA models, the majority of PPI models are not tailored for plant proteins; they are frequently trained on generalized datasets, which diminishes the transferability and biological validity of predictions when applied to plants. Besides PPI, machine learning is extensively employed to examine transcriptome alterations in response to viral infection. Gradient boosting and support vector machine (SVM) techniques facilitated the classification of expression profiles in *Solanum lycopersicum* infected with tomato spotted wilt virus (TSWV) and the identification of resistance-associated genes [107,108]. Genes associated with salicylic acid pathways and phytoalexin production, such as PR1 and EDS1, were recognized as crucial indicators. The incorporation of meteorological and physiological characteristics, including humidity and temperature, into the training set enhanced the model’s predictive accuracy to 89% [109,110]. Nevertheless, the validation of such models’ conclusions in planta is infrequent, and the anticipated markers do not consistently demonstrate functional activity in natural settings, particularly in field varieties and under complex stressors.

Convolutional neural networks are extensively employed for the visual diagnosis of viral infections, including CMV, TMV, and TYLCV [111,112]. Ashtagi (2024) [113] employed a hybrid CNN and Random Forest model to assess pictures of *N. tabacum* leaves infected with TMV. The model exhibited an accuracy of 95% and an F1-measure of 0.94 during testing, indicating resilience to fluctuations in external factors, such as lighting and leaf morphology. The focus is on predicting plant vulnerability to viruses using whole-genome data. In a model applied to the *Oryza sativa* system involving rice tungro bacilliform virus (RTBV), the Decision Tree and SVM algorithms facilitated the identification of allelic changes in the eIF(iso)4G gene, which were associated with susceptibility to infection [96,114]. This was further validated using CRISPR interference, facilitating the practical application of genetic resistance by altering this region.

Consequently, the utilization of machine learning in the examination of plant–virus systems has novel prospects for the precise discovery of essential molecular connections, including protein complexes and gene expression regulation networks. Nonetheless, the overarching difficulty persists: elevated efficiency in silico is not invariably validated in planta, particularly under circumstances where gene expression and activity are contingent upon environmental factors, soil composition, microbiome interactions, and the developmental stage of the plant. This necessitates the integration of forecasts with multivariate field experiments. As of yet, ML methods have demonstrated their utility in generating pathogenic models, evaluating breeding, and developing resistant types (Table 3). Nonetheless, extensive deployment of these methodologies necessitates additional growth and standardization of validation data, along with a robust combination of bioinformatic predictions and experimental validation.

### 2.4. Using Generative AI Models in CRISPR Design

The formulation of efficient antiviral methods for agricultural crops is gaining significance due to global climate change and rising biotic stressors [114]. Viral infections such as TYLCV, RTBV, CMV, and CLCuV result in substantial yearly crop losses [115,116,117]. The most effective strategy for addressing these infections is the application of CRISPR/Cas technology. In this respect, generative AI models like AlphaFold2, RoseTTAFold, and ESMFold, which facilitate the prediction of three-dimensional structures of proteins and protein–protein complexes with atomic precision, are notably noteworthy. These technologies are revolutionizing the methodology for creating CRISPR systems, especially for modifying plant genomes to confer resistance to viral infections.

As a result, AlphaFold2 was employed to predict the structure of the TYLCV Rep capsid protein and to pinpoint essential areas of its interaction with *N. benthamiana* host proteins. This facilitated the creation of efficient target sites for Cas12a, leading to a 70–80% decrease in viral replication in in planta tests [118,119]. These approaches support the rational identification of target regions within both viral genomes and plant susceptibility genes.

Another instance is employing RoseTTAFold and ProteinMPNN to engineer stable and highly specific Cas protein variants with enhanced PAM recognition, tailored for functionality in plant cells [120,121]. Researchers utilized ESMFold to create models of mutant variants of Cas13a that are effective against RNA viruses, including potato virus Y (PVY) and CMV, and tailored to the cellular environment of Solanum tuberosum. The anticipated structures were employed for in vitro assessments of binding and catalytic efficacy, facilitating the identification of variations with improved cytoplasmic stability and nuclear localization. Generative models are employed to examine interactions between viral and plant proteins. AlphaFold2 has been applied to predict the interaction between the VPg protein of rice yellow mottle virus (RYMV) and the eukaryotic translation initiation factor eIF4E in *Oryza sativa*, as demonstrated in previous studies [122,123]. Based on these structural predictions, targeted gene modifications were introduced via CRISPR/Cas9 to disrupt the interaction interface, resulting in enhanced viral resistance without compromising plant viability [124]. However, structure prediction tools such as AlphaFold2 and related models exhibit inherent limitations, as they do not account for the cellular environment, dynamic expression patterns, post-translational modifications, or the temporal behavior of protein interactions [68].

Notwithstanding these remarkable accomplishments, considerable limits persist. The majority of training data for AlphaFold2 and analogous models is sourced from animal and bacterial research, potentially constraining the precision of predictions for particular plant proteins and viruses. Secondly, the existing structural data on plant viruses remains insufficient, hindering model validation. In addition, the integration of generative models into applied biotechnology necessitates defined protocols, in-plant validation, and an assessment of plant physiological characteristics. The emergence of artificially designed proteins generated entirely de novo by AI-based platforms such as ProGen2 [125,126] raises biosafety concerns, as these molecules lack natural equivalents and may exhibit unpredictable behavior in biological contexts.

The implementation of generative AI models such as AlphaFold2, RoseTTAFold, and ESMFold demonstrates considerable promise for designing CRISPR system components targeting plant viruses (Table 4). These methods facilitate the synthesis of high-fidelity proteins and gRNAs customized for plant cell environments, hence advancing more predictable, efficient, and secure genome editing in future agriculture.

## 3. Conclusions

The integration of artificial intelligence into CRISPR-based technologies represents a transformative advancement in the management of plant viral infections. Advancements in deep learning, transformers, and generative models like AlphaFold2, RoseTTAFold, ESMFold, and ProGen2 have markedly enhanced the precision of designing guide RNAs, Cas proteins, and delivery vectors customized for specific plant systems and viral pathogens. These interventions have exhibited compelling efficacy in mitigating significant plant diseases, including TYLCV, RTBV, CMV, TMV, and CLCuV, in crops such as *Solanum lycopersicum*, *Oryza sativa*, *Gossypium hirsutum*, and *N. benthamiana*. AI-enabled approaches now enable the resolution of previously intractable challenges, such as molecular characterization of virus–host interactions, predictive modeling of CRISPR targeting specificity, prediction of off-target effects, and structural optimization of genome editing enzymes. The predictive accuracy of AI models often declines when transitioning from in vitro models to actual plants. Many algorithms fail to consider epigenetic changes, chromatin accessibility, and intertissue variations in gene expression. The application of generative AI models for creating Cas proteins with altered PAM recognition, increased stability in plant environments, and reduced off-target risks is a noteworthy area of scientific exploration. The application of these technologies enables the shift from universal to precise editing, which is essential given the fast evolution of viral infections.

Nonetheless, these potent instruments are linked to several biological and ethical dilemmas. At the biological level, there exists a risk of inadvertent disruption of plant genomes, encompassing off-target consequences, horizontal transmission of changed components, and unanticipated interactions with the microbiome and ecosystem. This is especially crucial for the utilization of generative models, which may generate unverified protein sequences necessitating obligatory multi-tier validation.

Ethical considerations pertain to the transparency of AI model applications, the interpretability of their predictions, and the ownership of algorithms, data, and resultant genetic structures. The development of proprietary AI systems driven by commercial interests may hinder equitable access to innovation for resource-constrained nations, particularly those vulnerable to widespread viral plant outbreaks. Moreover, there is a necessity for global standardization of legislation pertaining to gene-edited species, particularly with the application of AI models, which add a new layer of complexity in assessing the degree of genomic alteration. An interdisciplinary approach, integrating the expertise of professionals in molecular biology, bioinformatics, agronomy, ethics, and legislation, is especially crucial in this setting. Such collaboration is essential for the advancement of sustainable, scientifically robust, and socially acceptable plant editing methodologies utilizing AI-enhanced CRISPR systems. The creation of open and accessible tools is equally crucial to guarantee global equity and participation in scientific and technological advancement.

AI significantly improves CRISPR capabilities and fundamentally alters its promise in plant science, facilitating safer, more efficient, and more adaptable management of viral risks. Harnessing this potential requires not just technological advancements but also a carefully designed scientific, regulatory, and ethical framework that guarantees sustainability and fosters public trust in emerging biotechnologies.

## Figures and Tables

**Table 1:** Comparison of sgRNA prediction tools for CRISPR/Cas in plants.

Name	Goal	Model	Learning Type	+	—	Organisms	Metrics	Metrics (Plants)
sgRNACNN	Prediction of sgRNA efficiency	Hybrid CNN	Deep Learning (Keras/TensorFlow)	Trained on plant data, high accuracy	Domain sensitive, requires transfer learning	*A. thaliana*, *O. sativa*, *Z. mays*, *S. lycopersicum*	ROC-AUC ~0.85, Spearman ~0.70	ROC-AUC ~0.85, Spearman ~0.70
Positional classifiers	On-target activity	Gradient Boosting	Classical ML	Interpretability, low computational requirements	They do not take into account 3D chromatin and epigenetics.	*O. sativa*, *Z. mays*, *A. thaliana*	Spearman ~0.55	Spearman ~0.45–0.55
DeepCpf1	Prediction of Cas12a activity	CNN + FC Layers	Deep Learning (TensorFlow)	Accuracy for Cas12a	No plant data, reduced accuracy	*O. sativa*	ROC-AUC ~0.82, R^2^ ~0.65	AUC ~0.55–0.60
sgRNA Scorer 2.0	Predicting gRNA efficiency for Cas9 in different genomes	Gradient Boosting	Classical ML	Simplicity, accessibility	Heuristic model, no plant data	*O. sativa*, *N. benthamiana*	Correlation ~0.70	Correlation ~0.30–0.40
E-CRISP	sgRNA generation and scoring	Rule-based	Rule-based	Simplicity, speed	Does not take epigenetics into account	*A. thaliana*, *O. sativa*	Corr ~0.35	~0.30–0.35
CHOPCHOP	A universal web tool for sgRNA	Rule-based	Rule-based	Support for many Cas, simplicity	Primitive on-target metrics	*N. benthamiana*, *S. lycopersicum*, *O. sativa*	Corr ~0.30	Correlation ~0.30
Cas-OFFinder	Off-target search	Exhaustive search algorithm	no ML	Flexibility, PAM support, speed	No consideration of the biocontext	*Z. mays*, *G. max*, *N. benthamiana*	-	Greatly overestimates the risks
CRISPR Genome-wide analysis	Genomic screening	Pipeline-based	no ML	Genomic coverage	No ML, not adapted to plants	*C. annuum* L., *O. Sativa*, *A. thaliana*	-	-

**Table 2:** Application of AI to design Cas proteins against plant viruses.

Cas Protein	Target Virus	Plant	AI	Efficiency	+	—
FnCas9	CMV, TMV	*N. benthamiana*, *A. thaliana*	CNN for assessing FnCas9-RNA interactions	Decreased viral RNA levels, inherited	RNA viruses, no need for DNA editing	Potential off-target for RNA
SpCas9	CLCuMuV	*N. benthamiana*, *A. thaliana*	Deep-RPA (1D Convolutional Neural Network)	99% accuracy in predicting off-target sites	Targeting multiple sites	Risk of mosaic mutation, Limited to model plants
SaCas9 (modified)	TYLCV, TMV	*S. lycopersicum*, *N. benthamiana*	GUIDE-seq + Deep Learning (CNN for off-target)	Off-target reduction without loss of activity (~80–90%)	Improved specificity; suitable for vectors	Not tested in plants; obtained in animal models
CasRx (RfxCas13d)	ssRNA viruses	*N. benthamiana*	Attention network for RNA structure	Suppression of viral expression by more than 80%	High specificity, small size	HEPN domain activity
Cas13a (codon-optimized)	TuMV	*N. benthamiana*	DeepCodon (DL for codon optimization)	Cas13a expression increased 2.3-fold; viral load decreased	Enhanced expression through DL optimization	Limited to plants with PVX infection
Cas12a (modified)	TYLCV	*S.* *lycopersicum*	CRISPR-GAN (DL for generating Cas12a variants)	Cas12a activity increased by 40% compared to wild type	Extended PAM profiles	High computational costs

**Table 3:** Tools for analyzing virus–plant interactions.

Name	Type of Learning	Application	Plante	Target Virus	+	—	Metrics (Accuracy)
CBIL-VHPLI	CNN + BiLSTM + Transfer Learning	Prediction of viral protein PPIs from lncRNA	*G* *. hirsutum*	CLCuMB	High accuracy, generalizability, adaptability to new data	Requires large training samples, high computational load	Accuracy: 91.6%, Precision: ~93%
SVM + Gradient Boosting	ML, transcriptome classification	Determination of resistance markers at the expression level	*S.* *lycopersicum*	TSWV	Biomarker detection, integration with climate factors	Sensitive to sampling, requires annotated data	Accuracy: ~89%
CNN + Random Forest	Hybrid Deep Learning + ML	Classification of images of viral infection symptoms	*N. tabacum*	TMV	Resistance to visual noise, high F1 metric	Dependence on image quality and domain	Accuracy: 95%, F1: ~0.94
Decision Tree + SVM	ML on genomic SNP data	Detection of susceptibility alleles (eIF(iso)4G)	*O.* *sativa*	RTBV	Detection of functionally significant mutations	Further validation is needed (CRISPR)	F1: ~0.88

**Table 4:** Application of generative AI models for designing Cas proteins against plant viruses.

AI Model	Goal	Virus/Plant	Efficiency	+	—
AlphaFold2	Prediction of 3D structures of Cas proteins (Cas9, Cas12a) and TYLCV virus proteins for the selection of interaction interfaces	TYLCV/*S. lycopersicum*	RMSD < 2Å, high structural accuracy; used to create stable complexes with targeted mutations	Allows for clarification of Cas protein interactions with viral DNA, speeding up the design cycle	Does not predict time-scale dynamics; subsequent molecular dynamics simulations are required
RoseTTAFold	Engineering Cas13a for RNA viruses, modeling the complex structure of Cas13-viral RNA	RTBV/*O. sativa*	RMSD 1.8–2.3Å; the accuracy of the complex prediction has been confirmed experimentally	Accounting for intermolecular interfaces, support for multi-chain modeling	High computational load, limited training set of plant viruses
ProGen2	Generation of new Cas protein variants with altered PAM recognition	CLCuV/*G. hirsutum*	~30% of generated sequences retained functionality in vitro	The possibility of creating non-standard Cas proteins with new specificities	Low accuracy without filtering; further structure validation required
ESMFold (Meta AI)	Rapid structure prediction of modified Cas proteins to improve stability in plant cytoplasm	TMV/*N. tabacum*	MSD ~2.1Å; models were used to screen proteins with high resistance to degradation	high speed, no alignment required	No accurate accounting of interactions with DNA/RNA; requires supplementation with molecular dynamics

## Data Availability

No new data were created or analyzed in this study.
